# Is Ergothioneine an Important Source of Plasma Trimethylamine *N*-Oxide in Humans?

**DOI:** 10.3390/antiox15070819

**Published:** 2026-06-29

**Authors:** Irwin K. Cheah, Lik Hang Wu, Richard M. Y. Tang, Ulrike Rieprecht, Leroy Sivappiragasam Pakkiri, Arthur Mark Richards, Chester Lee Drum, Barry Halliwell

**Affiliations:** 1 Neurobiology Programme, Life Science Institute, National University of Singapore, Singapore 117456, Singapore; bchickm@nus.edu.sg (I.K.C.); bchmyrt@nus.edu.sg (R.M.Y.T.);; 2 Department of Biochemistry, Yong Loo Lin School of Medicine, National University of Singapore, Singapore 117596, Singapore; 3 Cardiovascular Research Institute, National University Health System, Singapore 117599, Singapore; likhang_wu@u.nus.edu (L.H.W.); mdclspk@nus.edu.sg (L.S.P.); mark.richards@nus.edu.sg (A.M.R.); mdccld@nus.edu.sg (C.L.D.); 4 Department of Medicine, Yong Loo Lin School of Medicine, National University of Singapore, Singapore 119228, Singapore; 5 Department of Data Science, School of Frontier Engineering, The Kitasato Institute, Kanagawa 252-0373, Japan

**Keywords:** ergothioneine, trimethylamine, trimethylamine-*N*-oxide, neurodegeneration, ergothionase, gut microbiota

## Abstract

High circulating levels of trimethylamine-*N*-oxide (TMAO), largely produced by hepatic oxidation of gut-microbiota-derived trimethylamine (TMA), are associated with increased risk of cardiometabolic and neurodegenerative diseases. In contrast, the diet-derived compound ergothioneine (ET) possesses cytoprotective and neuroprotective properties, and higher circulating ET levels have been linked to a lower risk of cardiovascular, neurodegenerative, and other age-related disorders. However, concerns have been raised that microbial degradation of ET may also contribute to the TMAO pool. In this study, we examined the relationship between ET and TMAO. Bioinformatic analyses indicated that ergothionase, the enzyme responsible for ET degradation to trimethylamine (TMA), is restricted to a limited number of bacterial genera and is far less prevalent than choline trimethylamine lyase, which generates TMA from choline. In a randomised, placebo-controlled human study, ET supplementation (25 mg/day for 7 days) significantly increased plasma ET levels but did not increase TMAO concentrations. Similarly, in a heart failure cohort, plasma ET showed no correlation with TMA or TMAO levels, whereas TMAO was clearly correlated with TMA. Collectively, these findings suggest that ET is unlikely to contribute significantly to systemic TMAO levels.

## 1. Introduction

The gut-microbiota-derived metabolite, trimethylamine-*N*-oxide (TMAO), can be generated from the breakdown of certain dietary constituents including L-carnitine, choline, lecithin (phosphatidylcholine), betaine, and L-ergothioneine (ET) to trimethylamine (TMA), which is subsequently oxidised in the liver by a flavin-containing mono-oxygenase to form TMAO [1,2,3,4,5,6,7,8]. The health impacts of TMAO have garnered significant attention since its blood levels are correlated with increased risk of a wide range of cardiometabolic (e.g., hypertension, atherosclerosis, ischemic stroke, heart failure, acute myocardial infarction, diabetes, metabolic syndrome) and neurodegenerative diseases (mild cognitive impairment, Alzheimer’s disease; AD and Parkinson’s disease; PD) [1,2,3,4,5,6,7,8]. Multiple studies in cell and animal models have highlighted the potential contributory role of TMAO in the initiation and progression of these diseases [1,2,3,4,5,6,7,8], although this remains somewhat controversial since a few studies suggest that TMAO may actually be protective or may be neither causative nor protective but merely a biomarker of disrupted homeostasis [4,5]. Specifically for neurodegeneration, higher levels of TMAO were observed in the cerebrospinal fluids of AD patients [6], which have been shown to promote oxidative damage, mitochondrial dysfunction, and apoptosis of hippocampal neurons in rodents [7,8]. Indeed, increased oxidative damage and altered bioenergetics are key drivers of AD and other neurodegenerative disorders [9,10,11,12]. TMAO has also been suggested to promote neuroinflammation through activation of glia and astrocytes [13], compromise blood–brain barrier integrity and function [14], and promote aggregation of amyloid-beta peptide and tau protein [15].

Gut-microbiota diversity and bacterial metabolites are influenced by a wide range of factors including lifestyle, diet, age, and disease, and dysbiosis or imbalance of the microorganisms in the gut have been implicated in both the pathogenesis and progression of many diseases. Metabolites produced by gut microbiota are key mediators of a bi-directional communication between the gut and the brain (i.e., proponents of the gut–brain axis). This includes metabolites such as short-chain and branched-chain fatty acids, phenylacetylglutamine, neurotransmitters or their activators, and also TMA/TMAO [16]. Indeed, altered gut microbiota in neurodegenerative diseases, which for example in PD may precede neurological symptoms by many years [17], have been suggested to elevate levels of TMAO. Increased TMAO levels could then promote neuroinflammation (through microglial activation), mitochondrial dysfunction and oxidative damage, and protein misfolding and aggregation in the brain. For example, TMAO exacerbated neuroinflammation via striatal and substantia nigra astrocyte activation in 1-methyl-4-phenyl-1,2,3,6-tetrahydropyridine (MPTP)-induced murine models of PD [13] and promoted α-synuclein misfolding and aggregation. Remarkably, antibiotic treatment of α-synuclein over-expressing mice (PD model) ameliorated PD pathology, while recolonising with microbiota from PD-patients exacerbated PD symptoms, suggesting that the gut microbiota are a key factor of motor deficits and neuroinflammation in PD [6].

Ergothioneine (ET) is a sulphur-containing derivative of histidine, which animals and humans obtain through dietary sources (most notably mushrooms but it is present in many foods at lower levels) [18]. Interest in ET has grown rapidly in recent years, fuelled by rapidly-accumulating evidence of its widespread cytoprotective and neuroprotective benefits and the strong association of lower plasma ET concentrations with increased incidence of neurological and other age-related chronic disorders (as reviewed in [19,20,21]). Lower circulating ET levels have been associated with the incidence of mild cognitive impairment and dementia [22,23,24], Parkinson’s disease [19,21,25], age-related macular degeneration [26], chronic kidney disease [27,28], cardiovascular disease [29], frailty indicators [30,31], and pre-eclampsia [32]. Moreover, complementing these clinical associations, a wide body of experimental evidence in cell and animal models demonstrates the cytoprotective and neuroprotective properties of ET, including its ability to counteract oxidative damage, inflammation, mitochondrial dysfunction, and other pathological mechanisms in a range of disease models [19,21,33,34,35,36]. As such ET has been described as a ‘longevity vitamin’ and a potential nutrient/nutraceutical in the promotion of healthy aging and reducing the risk of age-related disease [19,20,37].

ET has also been suggested to be a source of TMAO [38,39]. Indeed, the enzyme ergothionase, a trimethylamine lyase found in some microorganisms including *Treponema denticola* [40], *Burkholderia* sp. [41,42], *Agrobacterium radiobacter* [43], *Alcaligenes faecalis* [44], *Clostridium symbiosum* [45], and *Escherichia coli* [46], catalyses the breakdown of ET into TMA and thiourocanic acid, which possibly evolved to allow the use of ET as a nitrogen source. However, while early biochemical studies suggested the presence of ET-degrading activity in crude *E. coli* extracts [46], this remains questionable, as current genomic annotations for *E. coli* do not identify any functional ergothionase homologs. Moreover, this earlier study did not identify a specific gene or protein, and importantly, *E. coli* is widely used as a host strain for ET biosynthesis (e.g., [47]), with no evidence of significant ET degradation.

Clinical studies with ET have shown no adverse effects associated with supplementation [48,49,50,51]. Moreover the U.S. Food and Drug Administration has designated ET as GRAS (Generally Recognised As Safe; GRN734—Blue California; and GRN001270—Gene III; the latter at up to 150 mg per day), and the European Food Safety Authority has decided that ET is safe as a food supplement [52], even in pregnant women and infants [53]. Contrary to its established safety profile, the possibility that supplementation of ET may increase levels of TMAO has been raised, inferring that ET administration is a ‘double-edged’ sword [38]. This study examines the correlation of plasma ET levels with TMAO levels in both ET-supplemented individuals and cohorts of patients with heart failure to determine if indeed ET may contribute to the TMAO pool.

## 2. Materials and Methods

### 2.1. Chemicals and Reagents

L-ergothioneine, L-ergothioneine-d_9_ (ET-d_9_), hercynine, hercynine-d_9_, ergothioneine sulphonate, and S-methyl ET standards were provided by ERGOLD (Montreuil, France). GMP-certified encapsulated ET and placebo (microcrystalline cellulose) used in human uptake studies [48] were kindly provided by ERGOLD (formerly Tetrahedron). TMAO-d_9_ was purchased from Cambridge Isotope Laboratories (Tewksbury, MA, USA). TMA, TMAO, betaine and all other chemicals (unless otherwise stated) were purchased from Sigma-Aldrich (Saint Louis, MO, USA).

### 2.2. Administration of ET to Healthy Volunteers

De-identified plasma samples were from healthy young (21–35 y/o) male adult Chinese volunteers supplemented with either placebo or 25 mg ET as detailed in [48]. Informed consent was provided by all participants, and the study was approved by the National Healthcare Group Domain Specific Review Board (Protocol 2013/01074; 21 February 2014). Briefly, this double-blind, placebo-controlled study was designed to investigate the uptake and pharmacokinetics of ET following oral administration, through daily oral administration of placebo, or 25 mg ET (n = 15/arm) for 7 days with blood collected at baseline and days 3, 5 and 8. Venous blood was collected in EDTA-spray coated blood tubes (BD Vacutainer; Becton Dickinson, NJ, USA) and centrifuged at 2500 *g* for 15 min to separate plasma. Plasma aliquots were stored at −80 °C until analysis (ET is stable under these conditions). The levels of ET and its metabolites and TMAO were analysed in the plasma samples (n = 120) by liquid chromatography–mass spectrometry (LC-MS/MS).

### 2.3. Plasma Sample Preparation and Analysis

All samples were analysed on an Agilent 1290 Infinity II UPLC coupled to an Agilent 6460-QQQ electrospray ionization mass spectrometer (Agilent Technologies, Santa Clara, CA, USA). Plasma samples were prepared for LC-MS/MS analysis by adding 10 μL of plasma to 50 μL of ice-cold methanol containing heavy-labelled internal standards (2 μL of 25 μM ET-d_9_, 1 μL of 25 μM hercynine-d_9_, and 1 μL of TMAO-d_9_). Samples were vortexed and incubated overnight at −20 °C then vortexed and centrifuged at 4 °C and 14,000× *g* for 15 min. The supernatants were transferred to glass vials for drying under a stream of N_2_ gas at 35 °C. Samples were reconstituted in 100 μL of 90% methanol then diluted ten-fold with ultrapure water and transferred into silanized vial inserts for measurement by LC-MS/MS. Sample vials were maintained at 10 °C on the autosampler until analysis.

For analysis, 5 μL of extracted samples were injected onto a Cogent Diamond Hydride 1.0 column, which was 2.1 mm ID × 100 mm in length and 120 A 2.2 μm (Microsolv Technology Corp., Leland, NC, USA). Chromatographic separation was achieved using a gradient elution at 0.5 mL/min for 15 min. Solvent A was acetonitrile (HPLC-grade, Fisher Scientific, Waltham, MA, USA), containing 0.1% formic acid, and Solvent B was ultrapure water (Arium Pro, Sartorius, Gottingen, Germany) containing 0.1% formic acid. The gradient elution commenced with 90% Solvent A: 10% Solvent B for 2 min, followed by a gradual adjustment to 30% Solvent A: 70% Solvent B over 8 min, and finally 100% Solvent B for 2.5 min and re-equilibrated prior to the subsequent sample injection.

### 2.4. Ion Transitions and Mass Spectrometer Parameters

The precursor-to-product ion transitions and fragmentor voltages (V)/collision energies (eV) for each compound were as follows: ET: 230.1 → 186 and 106 V/9 eV; hercynine: 198.1 → 95.1 and 95 V/21 eV; ETSO_3_: 278 → 154 and 120 V/15 eV; S-methyl-ET: 244.1 → 141 and 92 V/17 eV; ET-d_9_: 239.1 → 195.1 and 110 V/10 eV; hercynine-d_9_: 207.2 → 95.1 and 95 V/22 eV; betaine: 117.9 → 59.4 and 110 V/20 eV; carnitine: 162 → 103 and 110 V/17 eV; choline: 104 → 60.4 and 100 V/18 eV; TMA: 60.4 → 45 and 95 V/14 eV; TMAO: 76 → 59 and 95 V/11 eV; and TMAO-d_9_: 85 → 68 and 95 V/11 eV.

### 2.5. Protein Basic Local Alignment Search Tool for Ergothionase

A search was conducted using the Protein Basic Local Alignment Search Tool (BLAST-P) through the National Centre for Biotechnology Information (NCBI), with the known ergothionase protein sequence from *Burkholderia* (accession no. BAM63550) as a query sequence. Searches were performed against the clustered NR database using default parameters.

### 2.6. Heart Failure Cohort

Plasma samples were drawn from two prospectively recruited heart failure (HF) cohorts: the Singapore Heart Failure Outcomes and Phenotypes (SHOP) study and the Prospective Evaluation of Outcome in Patients with Heart Failure with Preserved Left Ventricular Ejection Fraction (PEOPLE) study. All participants provided informed consent, and the study was approved by the SingHealth Centralised Institutional Review Board (Protocol No. CIRB: 2015/2194; 11 August 2015). Details of these cohorts are provided in Table 1 and have been described previously [54,55,56]. Briefly, the dataset comprised adult participants aged >18 years. To ensure that patient phenotypes, including metabolic profiles, biomarker measurements, and ejection fraction, were temporally aligned and reflected the chronic treated phase of HF, recruitment was conducted during the early chronic phase of HF, defined as within 6 months following a documented episode of HF decompensation leading to hospital admission. Patients were enrolled when clinically stable and judged fit for discharge, or at routine outpatient follow-up. Inclusion criteria were presentation to hospital with a primary diagnosis of HF, attendance at hospital clinics for HF management resulting in primary hospital admission, or attendance at outpatient clinics for HF management within 6 months of HF decompensation. Exclusion criteria included the inability to provide consent, inability to comply with study requirements, severe valvular disease, acute coronary syndrome, end-stage renal failure, constrictive pericarditis, isolated right heart failure, life-threatening comorbidity with an expected survival of less than 1 year, and participation in other clinical trials. Echocardiography-based assessment of left ventricular ejection fraction (LVEF) was performed, and the HF participants spanned the full LVEF spectrum, including HF with preserved ejection fraction (HFpEF; LVEF ≥ 50%), HF with mid-range ejection fraction (HFmrEF; 50% > LVEF ≥ 40%), and HF with reduced ejection fraction (HFrEF; 40% > LVEF). For the present analysis, only cases with complete clinical data and detectable baseline levels of ET, TMA, and TMAO, were included.

### 2.7. Metabolomics Measurement in the Heart Failure Cohort

Plasma concentrations of ET, TMA, and TMAO were extracted from an existing metabolomics dataset from the PEOPLE and SHOP cohorts. This dataset comprises concentration readouts from a predefined metabolite panel quantified by LC-MS/MS, using the same panel-based assay platform and underlying analytical workflow as in a previous outcome-association study of metabolite signatures [55]. Briefly, samples for metabolomic analyses were collected under non-fasting clinical conditions. Blood samples were centrifuged at 3000 rpm for 10 min at room temperature, and plasma aliquots were stored at −80 °C until analysis. Metabolites were extracted using acetonitrile containing 0.1% formic acid. A fixed concentration of internal standards was spiked into each sample, and analyte peak areas were normalised to the internal standards to account for technical variability, including fluctuations in instrument sensitivity and matrix effects across samples. The mobile phase consisted of solvent A, acetonitrile containing 0.1% formic acid, and solvent B, 20 mM ammonium formate at a pH of 4.0. An injection volume of 10 μL of extracted sample was analysed on an Agilent 1290 Infinity III LC. Chromatographic separation was achieved using a gradient elution of solvent B: 10% at 0 min, increased to 70% by 9.0 min and held until 11.0 min, then returned to 10% from 11.1 to 11.5 min, at a flow rate of 0.4 mL/min. Data acquisition was performed in dynamic multiple reaction monitoring mode using Agilent MassHunter acquisition software (version 10.0.127). Quality control measures removed unreliable measurements based on variability (CV ≤ 30%), consistency (D-ratio ≤ 50%), and signal strength (signal-to-noise ratio ≥ 3) thresholds. Only robust metabolites were retained across batches, and zero or infinite values were treated as missing before analysis.

### 2.8. Statistics

Data were tabulated using Microsoft Excel. Graphs were generated and statistical analyses (Pearson’s correlation coefficient and significance of the correlation) performed using GraphPad Prism (Version 10.3.1) and R (version 4.4.1). For the latter, a complete-case approach was taken, and only participants with complete data on all metabolites (ET, TMA, and TMAO) were included. Prior to analysis, metabolite levels were log-transformed and then z-score standardised. Pairwise Pearson’s correlations were computed between z-standardised ET, TMA, and TMAO levels. Differences between two independent groups were assessed using the Mann–Whitney U test. A two-sided *p*-value < 0.05 was considered statistically significant.

## 3. Results

### 3.1. Bioinformatic Detection of Ergothionase in Bacterial Genomes

By contrast to choline trimethylamine lyase (responsible for the generation of TMA from choline) which is abundantly found in many anaerobic gut microbes (19,824 protein database hits under bacteria; National Center for Biotechnology Information; NCBI), ergothioneine trimethylamine lyase (ergothionase) has only been definitively identified in a few microorganisms (namely *Burkholderia* sp., *Cupriavidus neocaledonicus*, *Cupriavidus taiwanensis*, *A. radiobacter*, and *T. denticola*; excluding activity in cell-free extracts).

**Figure 1:**
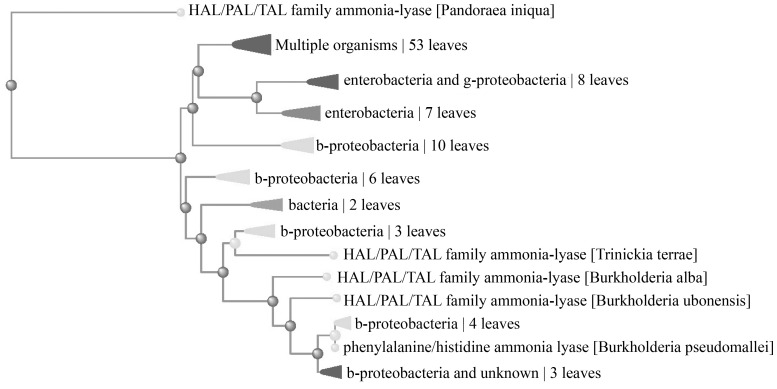
A BLAST-P search utilising the protein sequence of ergothionase from *Burkholderia pseudomallei* was performed with multiple hits on the structurally similar histidine ammonium lyase but not ergothionase.

In silico analyses using the protein sequence of ergothionase from *Burkholderia* were performed using the Protein Basic Local Alignment Search Tool (BLAST-P) and revealed only protein similarities to histidine, phenylalanine, and tyrosine ammonia lyases (HAL/PAL/TAL; Figure 1). Although these ammonia lyases are structurally similar, they are functionally distinct from ergothionase, which lacks the MIO (3,5-dihydro-5-methylidene-4H-imidazol-4-one) cofactor [40].

While further analyses are needed to firmly establish whether functionally analogous enzymes exist in other microbes, these data suggest that ergothionase is not widely distributed among gut microorganisms and is considerably less prevalent than choline trimethylamine lyase-containing microorganisms.

### 3.2. ET Supplementation Does Not Alter Plasma TMAO Levels

**Figure 2:**
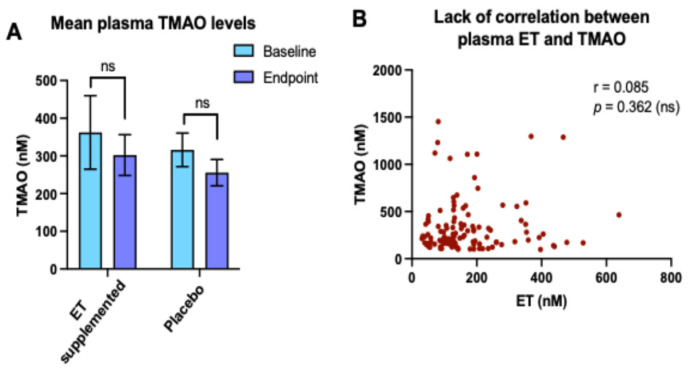
ET supplementation does not increase plasma TMAO levels, and TMAO and ET levels do not correlate. (**A**) Mean plasma TMAO levels (±SEM) at baseline and endpoint (day 8) in ET and placebo supplemented groups (n = 15/group). No significant difference in mean TMAO levels was observed at the endpoint relative to the baseline in both ET-supplemented and placebo groups. ns = not significant; Mann–Whitney U test. (**B**) No significant correlation was observed between plasma ET and TMAO levels both with and without ET supplementation; *p* = 0.362.

Supplementation of a healthy cohort of young Chinese males (refer Section 2.2) with 25 mg ET per day for 1 week led to a significant elevation in plasma and whole blood ET levels [48]. However, despite this increase, no significant change was observed in mean plasma TMAO levels following 7 days of supplementation (302.3 ± 54.0 nM), compared with baseline levels (362.5 ± 97.5 nM; *p* = 0.8702; Mann–Whitney test; Figure 2A). Similarly, no change in mean TMAO levels was observed between baseline and endpoint in the placebo group. Pearson’s correlation analysis of matched plasma samples further revealed no association between ET and TMAO levels (r = 0.085; *p* = 0.362; Figure 2B).

### 3.3. No Correlation of Plasma ET with TMA and TMAO in Heart Failure (HF) Patients

**Figure 3:**
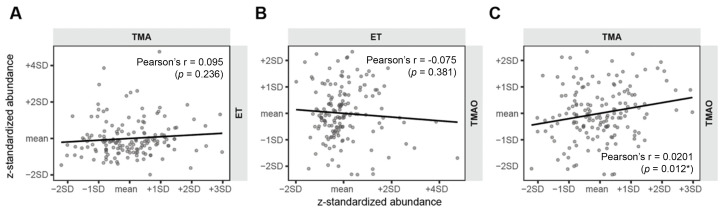
Correlations of plasma ET to (**A**) TMA and (**B**) TMAO, and (**C**) TMA to TMAO levels in HF patients. Pairwise Pearson’s correlations of plasma ET, TMA, and TMAO levels in HF patients (n = 157). All values were log-transformed, and the z-scores were standardised prior to analysis, with significance indicated by * *p* < 0.05.

Of the 157 HF cases (SHOP: n = 96; PEOPLE: n = 61), the majority were male (n = 134, 85.4%), Chinese (n = 74, 47.1%) and had HF-rEF (n = 136, 86.6%) with reduced ejection fraction. However, females and subjects of other ethnicities, including European, were also included (Table 1). Other baseline characteristics are summarised in Table 1. Pairwise Pearson’s correlations were computed between z-standardised plasma levels of ET, TMA, and TMAO in the 157 HF patients. ET levels were not correlated with either TMA (r = 0.095, *p* = 0.236; Figure 3A) or TMAO (r = −0.070, *p* = 0.381; Figure 3B) in the plasma of heart failure patients. However, TMA and TMAO showed a positive correlation (r = 0.201, *p* = 0.012; Figure 3C), consistent with TMAO being the downstream oxidation product of TMA.

### 3.4. TMAO Is Higher in HF as Compared to Control Cases but Not ET

**Figure 4:**
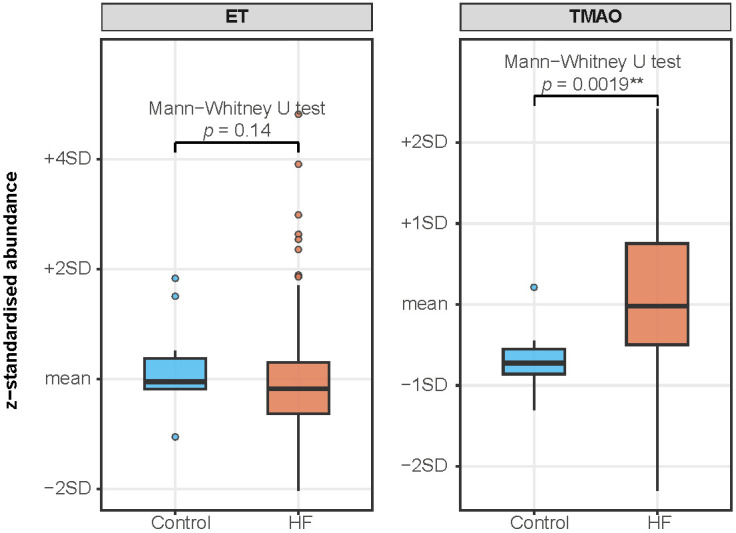
Boxplots of plasma ET and TMAO abundance in HF and non-HF control. Plasma levels of ET and TMAO were compared between 157 HF cases and 12 non-HF controls. Boxes indicate the interquartile range (IQR); the horizontal line denotes the median; whiskers extend to 1.5 × IQR. Between-group differences were assessed by two-sided Mann–Whitney U test; ** *p* < 0.005.

We also compared TMAO and ET levels between the 157 HF cases and 12 non-HF control cases in the same dataset (non-HF population attributes provided in Supplementary Table S1). Plasma TMAO was significantly higher in HF cases relative to non-HF controls (Mann–Whitney U test, *p* = 0.0019; Figure 4). By contrast, plasma ET did not differ significantly between HF cases and non-HF controls (Mann–Whitney U test, *p* = 0.14; Figure 4), consistent with the absence of correlation between ET and the TMAO observed above.

## 4. Discussion

Multiple epidemiological studies have linked low plasma or blood ET levels with an increased risk of cardiovascular disease [29], mild cognitive impairment [23,57], dementia [23,24], Parkinson’s disease [19,25], age-related macular degeneration [26], chronic kidney disease [27], pre-eclampsia [32], and frailty [30]. Although causality has not yet been fully established, extensive experimental evidence supports the cytoprotective and neuroprotective properties of ET (as reviewed in [19,20]), while early clinical studies suggest potential benefits of ET supplementation on cognitive and sleep health and demonstrate a strong safety profile [49,51]. Mechanistically, ET has been shown to exert its neuroprotective effects through multiple pathways, including the attenuation of oxidative stress and inflammation, preservation of mitochondrial function, protection against amyloid oligomer toxicity, and potential modulation of neurogenesis through promotion of neurotrophic factors and neuronal differentiation [58,59,60].

Despite the well-established beneficial and protective properties of ET, it has been suggested that ET supplementation may represent ‘a double-edge sword’ [38,39], due to the potential for gut microbial metabolism of ET to TMA, and subsequently TMAO. However, the present study demonstrates no correlation between plasma ET and TMAO levels in healthy Chinese volunteers supplemented with ET (25 mg per day for one week). Similarly, analysis of a cohort of heart failure patients revealed no association between ET and TMA or TMAO levels in plasma (Figure 3). Moreover, plasma TMAO, but not ET levels, were significantly elevated in heart failure patients compared with healthy controls, reinforcing the lack of association between the two metabolites (Figure 4). We acknowledge that the ET supplementation studies were limited to a Chinese cohort with short-term supplementation (7 days) and the limited number and differences in ethnicity of healthy controls for comparison with the HF cohort, possibly giving rise to differences in gut microbiota composition due to ethnicities. However, Zajac et al. [51] showed that longer-term ET supplementation (16-week administration of 10–25 mg ET) similarly did not increase plasma TMAO levels in a predominantly Caucasian (Australian) cohort [51], which further validates our findings.

In silico analyses indicate that ergothionase, responsible for the breakdown of ET to TMA, is only present in a few bacterial genera, limiting the likelihood of ET catabolism in the gut. However, this analysis does not account for poorly annotated or divergent homologues of ergothionase, and a deeper analysis may be needed to confirm the rarity of ergothionase activity. Interestingly, no ergothionase activity was detected in crude extracts of *Burkholderia* cultured in standard Luria-Bertani medium; however, when grown in media with ET as the sole nitrogen source, ergothionase was expressed [41], indicating that its activity is induced under specific nutrient conditions, which may not be prevalent in the human gut. In contrast to ergothionase, the choline trimethylamine lyase was found in many microorganisms and is widely distributed among gut microbes. Moreover, estimated dietary intakes of carnitine and choline, based on data from the NHANES (National Health and Nutrition Examination Survey, U.S.), are approximately 24–145 mg/day and ~400 mg/day [61], respectively, substantially higher than the estimated 1–5 mg/day intake of ET [62] and even supplements of ET (25 mg per day for a week; Figure 2) or 10–25 mg of ET per day for 16 weeks [51], which did not raise TMAO levels. Interestingly, both acetyl-L-carnitine and phosphatidylcholine supplementation have been suggested to slow cognitive decline and neurodegeneration [63,64]. However, amyotrophic lateral sclerosis patients supplemented with acetyl-L-carnitine have been reported to exhibit higher circulating levels of TMAO compared to age-matched controls [65], highlighting the need for further studies to clarify the metabolic consequences of these interventions and the potential implications of their gut-microbiota-derived metabolites. Other papers have identified carnitine as a likely major source of TMAO in rats and humans [66,67]. Taken together, these findings suggest that ET is unlikely to contribute significantly, if at all, to TMAO levels in the body.

However, the interaction between ET and the gut microbiota remains an area of considerable interest, particularly in relation to the compounds mediating the gut–brain axis [60]. ET has previously been proposed as a microbial mediator of gut–brain signalling, although this is unlikely to involve TMA. While there is little evidence that gut microbes are able to synthesise ET [60,68], many microorganisms possess ABC transporters that may facilitate ET uptake and accumulation [39,69]. For example, *Limosilactobacillus* (*Lactobacillus*) *reuteri* can avidly take up and retain ET from its environment [68], demonstrating that gut microbiota might influence host handling and bioavailability of dietary ET. Therefore, it is plausible that changes in gut microbial compositions, such as those reported in AD and PD, could impact ET uptake, metabolism, or retention in the host and thereby contribute to lower circulating ET levels observed in these neurodegenerative conditions. A recent study by Zheng et al. [70] found that antipsychotic therapies decrease ET levels, and this is suggested to be due to depletion of ET-producing *Cyanobacteria* species in the gut. However, bacteria from this phyla represent a minority of the gut microbiota, and there is little evidence to suggest that they produce ET in the gut (reviewed in [60]). Curiously, supplementation of mice with ET producing *Cyanobacteria* spp. and *Synechococcus* spp. was shown to limit olanzapine-induced cognitive impairment in the animals [70].

In addition, little is known about whether gut microbiota generate ET-derived metabolites, other than TMA, or whether such metabolites contribute to gut–brain signalling. Interactions between dietary compounds, gut microbiota, and the brain are highly complex, and the effects of any metabolite are likely to depend on the microbial composition and host factors, including diet, dysbiosis, inflammatory state, and disease-associated metabolic changes. These factors may influence circulating TMAO [67] and other microbiota-associated metabolites, making direct causal relationships between any individual dietary compound and disease risk, difficult.

Alterations in the gut microbiota have also been implicated in the pathogenesis of other chronic diseases. Indeed, elevated levels of *Clostridium symbiosum*, which has been reported to possess ergothionase activity, have been observed in faecal samples from colorectal cancer patients [45]. In parallel, ET has been shown to induce necroptosis in colorectal cancer cells [71], perhaps suggesting that *C. symbiosum*-driven breakdown or depletion of ET may contribute to tumour progression. Furthermore, thiourocanic acid, another product of ET degradation alongside TMA, has been shown to support the growth and ATP synthesis of anaerobic gut bacteria such as *Bacteroides xylanisolvens*, through metabolic cross-feeding [45]. Feng et al. [72] further demonstrated that thiourocanate desulfhydrase, an enzyme present in a range of anaerobic gut bacteria, can catalyse the breakdown of thiourocanic acid to release hydrogen sulphide (H_2_S). However, these pathways are ultimately dependent on degradation of ET by ergothionase, which may be limited since ET supplementation does not appear to contribute to the TMAO pool.

ET’s strong safety profile is supported by toxicology studies in rats, which demonstrate no evidence of toxicity following administration of doses up to 1600 mg ET/kg body weight per day for 90 days [73]. Similarly, no adverse reactions have been reported in clinical trials to date, and supplementation in elderly subjects for up to 12 months has been well tolerated [49,51]. Further supporting this safety, the present study indicates that ET is not a major contributor to systemic TMAO levels, and in silico findings further suggest that ergothionase is not widely distributed in gut microbiota. Taken together, these findings reinforce the case for ET as a promising neuroprotective compound and indicate that ET supplementation is unlikely to make a meaningful contribution to systemic TMAO levels. Nevertheless, the broader association between ET and the gut microbiota, particularly in the context of gut–brain axis signalling and disease, warrants further investigation [60] and, of course, conclusions might be different in other populations.

## 5. Conclusions

In summary, our findings provide no evidence that dietary ET contributes significantly to the systemic TMAO pool, addressing a key concern regarding ET’s therapeutic use. Together with its favourable safety profile and well-established cytoprotective and neuroprotective properties, ET remains a promising candidate for treatment or prevention of age-related and neurodegenerative disorders. Nevertheless, the relationship between ET and the gut microbiota is likely to be more complex than TMA/TMAO generation alone, and further studies are needed to clarify how microbial uptake, metabolism, and gut–brain axis interactions may influence how ET impacts in human health or vice versa [60].

## Figures and Tables

**Table 1:** Baseline characteristics of the study population (n = 157): Values are median (interquartile range) for continuous and n (%) for categorical variables. pEF: preserved ejection fraction; rEF: reduced ejection fraction; LVEF: left ventricular ejection fraction; COPD: chronic obstructive pulmonary disease; ACEi/ARB: ACE inhibitors and angiotensin receptor blockers; MRA: mineralocorticoid receptor antagonists.

Demographics	
Age (years)	62.2 (53.1–71.2)
Female, n (%)	23 (14.6%)
BMI (kg/m^2^)	26.6 (23.4–31.2)
**Ethnicity**	
Chinese	74 (47.1%)
European	28 (17.8%)
Malay	20 (12.7%)
Maori	9 (5.7%)
Other	26 (16.6%)
**Heart failure characteristics**	
**HF subtypes**	
HF-pEF	21 (13.4%)
HF-rEF	136 (86.6%)
**NYHA class**	
I	29 (18.6%)
II	88 (56.4%)
III	33 (21.2%)
IV	6 (3.8%)
LVEF (%)	28.8 (20–39.4)
Systolic BP (mmHg)	116.5 (102.8–130.2)
Diastolic BP (mmHg)	68.5 (60–80)
Heart rate (bpm)	75.5 (64–85)
**Laboratory values**	
Plasma creatinine (μmol/L)	107 (84–143)
**Comorbidities**	
Diabetes mellitus, n (%)	89 (56.7%)
Hypertension, n (%)	107 (68.2%)
COPD, n (%)	23 (14.6%)
Atrial fibrillation, n (%)	62 (39.5%)
Coronary artery disease, n (%)	93 (60.4%)
**Medications at baseline**	
Beta-blocker, n (%)	138 (87.9%)
ACEi/ARB, n (%)	122 (77.7%)
MRA, n (%)	80 (51%)
Diuretic, n (%)	149 (94.9%)

## Data Availability

The data presented in this study are available from the corresponding author upon reasonable request.

## Supplementary Materials

The following supporting information can be downloaded at https://www.mdpi.com/article/10.3390/antiox15070819/s1, Table S1: baseline characteristics of the control population (n = 12) for comparison of TMAO levels.

## Abbreviations

The following abbreviations are used: AD—Alzheimer’s disease; ET—ergothioneine; HF—heart failure; LC-MS/MS—liquid chromatography mass spectrometry; LVEF—left ventricular ejection fraction; MPTP—1-methyl-4-phenyl-1,2,3,6-tetrahydropyridine; PEOPLE—Prospective Evaluation of Outcome in Patients with Heart Failure with Preserved Left Ventricular Ejection Fraction study; PD—Parkinson’s disease; SHOP—Singapore Heart Failure Outcomes and Phenotypes study; TMA—trimethylamine; TMAO—trimethylamine-*N*-oxide; and GRAS—Generally Recognised as Safe.

## References

[1] Ren Z., Mo L. (2025). Association between levels of trimethylamine N-oxide and cognitive dysfunction: A systematic review and meta-analysis. PeerJ.

[2] Chung S.J., Rim J.H., Ji D., Lee S., Yoo H.S., Jung J.H., Baik K., Choi Y., Ye B.S., Sohn Y.H. (2021). Gut microbiota-derived metabolite trimethylamine N-oxide as a biomarker in early Parkinson’s disease. Nutrition.

[3] Quan W., Qiao C.M., Niu G.Y., Wu J., Zhao L.P., Cui C., Zhao W.J., Shen Y.Q. (2023). Trimethylamine N-Oxide Exacerbates Neuroinflammation and Motor Dysfunction in an Acute MPTP Mice Model of Parkinson’s Disease. Brain Sci..

[4] Nowiński A., Ufnal M. (2018). Trimethylamine N-oxide: A harmful, protective or diagnostic marker in lifestyle diseases?. Nutrition.

[5] Videja M., Vilskersts R., Korzh S., Cirule H., Sevostjanovs E., Dambrova M., Makrecka-Kuka M. (2020). Microbiota-Derived Metabolite Trimethylamine N-Oxide Protects Mitochondrial Energy Metabolism and Cardiac Functionality in a Rat Model of Right Ventricle Heart Failure. Front. Cell Dev. Biol..

[6] Vogt N.M., Romano K.A., Darst B.F., Engelman C.D., Johnson S.C., Carlsson C.M., Asthana S., Blennow K., Zetterberg H., Bendlin B.B. (2018). The gut microbiota-derived metabolite trimethylamine N-oxide is elevated in Alzheimer’s disease. Alzheimers Res. Ther..

[7] Deng Y., Zou J., Hong Y., Peng Q., Fu X., Duan R., Chen J., Chen X. (2022). Higher Circulating Trimethylamine N-Oxide Aggravates Cognitive Impairment Probably via Downregulating Hippocampal SIRT1 in Vascular Dementia Rats. Cells.

[8] Li D., Ke Y., Zhan R., Liu C., Zhao M., Zeng A., Shi X., Ji L., Cheng S., Pan B. (2018). Trimethylamine-N-oxide promotes brain aging and cognitive impairment in mice. Aging Cell.

[9] Butterfield D.A., Halliwell B. (2019). Oxidative stress, dysfunctional glucose metabolism and Alzheimer disease. Nat. Rev. Neurosci..

[10] Butterfield D.A., Drake J., Pocernich C., Castegna A. (2001). Evidence of oxidative damage in Alzheimer’s disease brain: Central role for amyloid beta-peptide. Trends Mol. Med..

[11] Butterfield D.A. (2023). Oxidative Stress in Brain in Amnestic Mild Cognitive Impairment. Antioxidants.

[12] Perluigi M., Di Domenico F., Butterfield D.A. (2024). Oxidative damage in neurodegeneration: Roles in the pathogenesis and progression of Alzheimer disease. Physiol. Rev..

[13] Qiao C.M., Quan W., Zhou Y., Niu G.Y., Hong H., Wu J., Zhao L.P., Li T., Cui C., Zhao W.J. (2023). Orally Induced High Serum Level of Trimethylamine N-oxide Worsened Glial Reaction and Neuroinflammation on MPTP-Induced Acute Parkinson’s Disease Model Mice. Mol. Neurobiol..

[14] Hoyles L., Pontifex M.G., Rodriguez-Ramiro I., Anis-Alavi M.A., Jelane K.S., Snelling T., Solito E., Fonseca S., Carvalho A.L., Carding S.R. (2021). Regulation of blood-brain barrier integrity by microbiome-associated methylamines and cognition by trimethylamine N-oxide. Microbiome.

[15] Buawangpong N., Pinyopornpanish K., Siri-Angkul N., Chattipakorn N., Chattipakorn S.C. (2022). The role of trimethylamine-N-Oxide in the development of Alzheimer’s disease. J. Cell Physiol..

[16] Ahmed H., Leyrolle Q., Koistinen V., Karkkainen O., Laye S., Delzenne N., Hanhineva K. (2022). Microbiota-derived metabolites as drivers of gut-brain communication. Gut Microbes.

[17] Yang D., Zhao D., Ali Shah S.Z., Wu W., Lai M., Zhang X., Li J., Guan Z., Zhao H., Li W. (2019). The Role of the Gut Microbiota in the Pathogenesis of Parkinson’s Disease. Front. Neurol..

[18] Borodina I., Kenny L.C., McCarthy C.M., Paramasivan K., Pretorius E., Roberts T.J., van der Hoek S.A., Kell D.B. (2020). The biology of ergothioneine, an antioxidant nutraceutical. Nutr. Res. Rev..

[19] Halliwell B., Cheah I. (2024). Are age-related neurodegenerative diseases caused by a lack of the diet-derived compound ergothioneine?. Free Radic. Biol. Med..

[20] Halliwell B., Tang R.M.Y., Cheah I.K. (2023). Diet-Derived Antioxidants: The Special Case of Ergothioneine. Annu. Rev. Food Sci. Technol..

[21] Tng T.J.W., Cheah I.K., Halliwell B., Lim K.-L. (2026). Potential Protection Against Parkinson’s Disease by Ergothioneine—Nature’s Multifactorial Neuroprotectant. Antioxidants.

[22] Cheah I., Feng L., Tang R.M.Y., Lim K.H.M., Halliwell B. (2016). Ergothioneine levels in an elderly population decrease with age and incidence of cognitive decline; a risk factor for neurodegeneration?. Biochem. Biophys. Res. Commun..

[23] Wu L.Y., Cheah I.K., Chong J.R., Chai Y.L., Tan J.Y., Hilal S., Vrooman H., Chen C.P., Halliwell B., Lai M.K.P. (2021). Low plasma ergothioneine levels are associated with neurodegeneration and cerebrovascular disease in dementia. Free Radic. Biol. Med..

[24] Wu L.Y., Kan C.N., Cheah I.K., Chong J.R., Xu X., Vrooman H., Hilal S., Venketasubramanian N., Chen C.P., Halliwell B. (2022). Low Plasma Ergothioneine Predicts Cognitive and Functional Decline in an Elderly Cohort Attending Memory Clinics. Antioxidants.

[25] Hatano T., Saiki S., Okuzumi A., Mohney R.P., Hattori N. (2016). Identification of novel biomarkers for Parkinson’s disease by metabolomic technologies. J. Neurol. Neurosurg. Psychiatry.

[26] Cheah I.K., Fong Z.W., Chen L., Tang R.M.Y., Zhou L., Yanagi Y., Cheng C.-Y., Su X., Li X., Teo K.Y.C. (2026). Ergothioneine as a potential protective agent against macular degeneration and other eye disorders. Sci. Rep..

[27] Shinozaki Y., Furuichi K., Toyama T., Kitajima S., Hara A., Iwata Y., Sakai N., Shimizu M., Kaneko S., Isozumi N. (2017). Impairment of the carnitine/organic cation transporter 1-ergothioneine axis is mediated by intestinal transporter dysfunction in chronic kidney disease. Kidney Int..

[28] Suba J.K., Keo L.S., Sirich T.L. (2025). Depletion by Hemodialysis of the Antioxidant Ergothioneine. Kidney360.

[29] Smith E., Ottosson F., Hellstrand S., Ericson U., Orho-Melander M., Fernandez C., Melander O. (2020). Ergothioneine is associated with reduced mortality and decreased risk of cardiovascular disease. Heart.

[30] Kameda M., Teruya T., Yanagida M., Kondoh H. (2020). Frailty markers comprise blood metabolites involved in antioxidation, cognition, and mobility. Proc. Natl. Acad. Sci. USA.

[31] Kondoh H., Teruya T., Kameda M., Yanagida M. (2022). Decline of ergothioneine in frailty and cognition impairment. FEBS Lett..

[32] Kenny L.C., Brown L.W., Ortea P., Tuytten R., Kell D.B. (2023). Relationship between the concentration of ergothioneine in plasma and the likelihood of developing pre-eclampsia. Biosci. Rep..

[33] Tng T.J.W., Leow D.M.K., Goh G., Wang Z., Liu Y.M., Tang R.M.Y., Lai K.C.L., Basil A.H., Chong H.C., Goh W.W.B. (2025). Ergothioneine treatment ameliorates the pathological phenotypes of Parkinson’s disease models. J. Neurochem..

[34] Leow D.M., Cheah I.K., Chen L., Ng Y.K., Yeo C.J., Halliwell B., Ong W.Y. (2024). Ergothioneine-Mediated Neuroprotection of Human iPSC-Derived Dopaminergic Neurons. Antioxidants.

[35] Petrovic D., Slade L., Paikopoulos Y., D’Andrea D., Savic N., Stancic A., Miljkovic J.L., Vignane T., Drekolia M.K., Mladenovic D. (2025). Ergothioneine improves healthspan of aged animals by enhancing cGPDH activity through CSE-dependent persulfidation. Cell Metab..

[36] Sprenger H.G., Mittenbühler M.J., Sun Y., Van Vranken J.G., Schindler S., Jayaraj A., Khetarpal S.A., Smythers A.L., Vargas-Castillo A., Puszynska A.M. (2025). Ergothioneine controls mitochondrial function and exercise performance via direct activation of MPST. Cell Metab..

[37] Ames B.N. (2018). Prolonging healthy aging: Longevity vitamins and proteins. Proc. Natl. Acad. Sci. USA.

[38] Nielsen J. (2022). Bioactive metabolites: The double-edged sword in your food. Cell.

[39] Dumitrescu D.G., Gordon E.M., Kovalyova Y., Seminara A.B., Duncan-Lowey B., Forster E.R., Zhou W., Booth C.J., Shen A., Kranzusch P.J. (2022). A microbial transporter of the dietary antioxidant ergothioneine. Cell.

[40] Maurer A., Leisinger F., Lim D., Seebeck F.P. (2019). Structure and Mechanism of Ergothionase from Treponema denticola. Chemistry.

[41] Muramatsu H., Matsuo H., Okada N., Ueda M., Yamamoto H., Kato S., Nagata S. (2013). Characterization of ergothionase from Burkholderia sp. HME13 and its application to enzymatic quantification of ergothioneine. Appl. Microbiol. Biotechnol..

[42] Medellin B.P., Wang S., Liu P., Zhang Y.J. (2018). Structure and Function of Ergothionase, an Ergothioneine TMA-Lyase from the Soil Bacteria Burkholderia sp. HME13. FASEB J..

[43] Yan Q., Huang H., Zhang X. (2022). In Vitro Reconstitution of a Bacterial Ergothioneine Sulfonate Catabolic Pathway. ACS Catal..

[44] Kelly B., Appleman M.D. (1961). Degradation of ergothioneine by cell-free extracts of Alcaligenes faecalis. J. Bacteriol..

[45] Zhou Z., Jiang A., Jiang X., Hatzios S.K. (2025). Metabolic cross-feeding of a dietary antioxidant enhances anaerobic energy metabolism by human gut bacteria. Cell Host Microbe.

[46] Wolff J.B. (1962). Ergothionase from *Escherichia coli*. J. Biol. Chem..

[47] Zhang X., Guan J., Yang Y., Zuo S., Liu C., Wang P. (2026). Construction of an *E. coli* cell factory for ergothioneine through SAM-cycle enhancement and pathway reconstruction. J. Biotechnol..

[48] Cheah I.K., Tang R.M.Y., Yew T.S., Lim K.C., Halliwell B. (2017). Administration of pure ergothioneine to healthy human subjects; Uptake, metabolism and effects on biomarkers of oxidative damage and inflammation. Antioxid. Redox Signal.

[49] Yau Y.F., Cheah I.K., Mahendran R., Tang R.M., Chua R.Y., Goh R.E., Feng L., Li J., Kua E.H., Chen C. (2024). Investigating the efficacy of ergothioneine to delay cognitive decline in mild cognitively impaired subjects: A pilot study. J. Alzheimers Dis..

[50] Zajac I., Kakoschke N., May-Zhang L. (2024). The Effect of Ergothioneine Supplementation on Cognitive Function and Other Health-Related Outcomes in Older Adults With Subjective Memory Complaints. Curr. Dev. Nutr..

[51] Zajac I.T., Kakoschke N., Kuhn-Sherlock B., May-Zhang L.S. (2025). The Effect of Ergothioneine Supplementation on Cognitive Function, Memory, and Sleep in Older Adults with Subjective Memory Complaints: A Randomized Placebo-Controlled Trial. Nutraceuticals.

[52] Turck D., Bresson J.L., Burlingame B., Dean T., Fairweather-Tait S., Heinonen M., Hirsch-Ernst K.I., Mangelsdorf I., McArdle H.J., Naska A. (2016). Safety of synthetic l-ergothioneine (Ergoneine^®^) as a novel food pursuant to Regulation (EC) No 258/97. EFSA J..

[53] Turck D., Bresson J.L., Burlingame B., Dean T., Fairweather-Tait S., Heinonen M., Hirsch-Ernst K.I., Mangelsdorf I., McArdle H.J., Naska A. (2017). Statement on the safety of synthetic l-ergothioneine as a novel food—Supplementary dietary exposure and safety assessment for infants and young children, pregnant and breastfeeding women. EFSA J..

[54] Lam C.S.P., Gamble G.D., Ling L.H., Sim D., Leong K.T.G., Yeo P.S.D., Ong H.Y., Jaufeerally F., Ng T.P., Cameron V.A. (2018). Mortality associated with heart failure with preserved vs. reduced ejection fraction in a prospective international multi-ethnic cohort study. Eur. Heart J..

[55] Wu L.H., Pakkiri L.S., Tromp J., Lim P.L., Liu X.Y., Chan S.P., Yang J., Samant R., Dong J., Goh E. (2026). Differential metabolomic prediction of outcomes in HFpEF and HFrEF: A prospective international multi-ethnic cohort study. Cardiovasc. Res..

[56] Santhanakrishnan R., Ng T.P., Cameron V.A., Gamble G.D., Ling L.H., Sim D., Leong G.K., Yeo P.S., Ong H.Y., Jaufeerally F. (2013). The Singapore Heart Failure Outcomes and Phenotypes (SHOP) study and Prospective Evaluation of Outcome in Patients with Heart Failure with Preserved Left Ventricular Ejection Fraction (PEOPLE) study: Rationale and design. J. Card. Fail..

[57] Feng L., Cheah I.K., Ng M.M., Li J., Chan S.M., Lim S.L., Mahendran R., Kua E.H., Halliwell B. (2019). The Association between Mushroom Consumption and Mild Cognitive Impairment: A Community-Based Cross-Sectional Study in Singapore. J. Alzheimers Dis..

[58] Ishimoto T., Nakamichi N., Hosotani H., Masuo Y., Sugiura T., Kato Y. (2014). Organic cation transporter-mediated ergothioneine uptake in mouse neural progenitor cells suppresses proliferation and promotes differentiation into neurons. PLoS ONE.

[59] Ishimoto T., Masuo Y., Kato Y., Nakamichi N. (2019). Ergothioneine-induced neuronal differentiation is mediated through activation of S6K1 and neurotrophin 4/5-TrkB signaling in murine neural stem cells. Cell Signal.

[60] Halliwell B. (2026). Ergothioneine as a Protective Agent Against Age-Related Diseases: Issues Needing More Research. Preprints.

[61] Centers for Disease Control and Prevention (CDC), National Center for Health Statistics (NCHS) (2026). National Health and Nutrition Examination Survey Data.

[62] Beelman R., Kalaras M., Phillips A., Richie J. (2020). Is ergothioneine a ‘longevity vitamin’ limited in the American diet?. J. Nutr. Sci..

[63] Pennisi M., Lanza G., Cantone M., D’Amico E., Fisicaro F., Puglisi V., Vinciguerra L., Bella R., Vicari E., Malaguarnera G. (2020). Acetyl-L-Carnitine in Dementia and Other Cognitive Disorders: A Critical Update. Nutrients.

[64] Ylilauri M.P.T., Voutilainen S., Lönnroos E., Virtanen H.E.K., Tuomainen T.P., Salonen J.T., Virtanen J.K. (2019). Associations of dietary choline intake with risk of incident dementia and with cognitive performance: The Kuopio Ischaemic Heart Disease Risk Factor Study. Am. J. Clin. Nutr..

[65] Sotgia S., Zinellu A., Zoroddu S., Pateri M.I., Loi E., Pisano A., Sabalic A., Tutedde D., Vega-Benedetti A.F., Floris F. (2026). Elevated serum trimethylamine N-oxide (TMAO) and trimethyllysine in patients with amyotrophic lateral sclerosis (ALS): An exploratory case-control study. IBRO Neurosci. Rep..

[66] Kuka J., Liepinsh E., Makrecka-Kuka M., Liepins J., Cirule H., Gustina D., Loza E., Zharkova-Malkova O., Grinberga S., Pugovics O. (2014). Suppression of intestinal microbiota-dependent production of pro-atherogenic trimethylamine N-oxide by shifting L-carnitine microbial degradation. Life Sci..

[67] Manor O., Zubair N., Conomos M.P., Xu X., Rohwer J.E., Krafft C.E., Lovejoy J.C., Magis A.T. (2018). A Multi-omic Association Study of Trimethylamine N-Oxide. Cell Rep..

[68] Cheah I.K., Lee J.Z., Tang R.M.Y., Koh P.W., Halliwell B. (2022). Does Lactobacillus reuteri influence ergothioneine levels in the human body?. FEBS Lett..

[69] Zhang Y., Gonzalez-Gutierrez G., Legg K.A., Walsh B.J.C., Pis Diez C.M., Edmonds K.A., Giedroc D.P. (2022). Discovery and structure of a widespread bacterial ABC transporter specific for ergothioneine. Nat. Commun..

[70] Zheng M., Yan H., Hao W., An H., Chen X., Wu Q., Ge X., Ye H., Zhou M., Zhou G. (2026). Gut microbiota-derived ergothioneine alleviates antipsychotic-induced synaptic and cognitive impairments. Cell Host Microbe.

[71] D’Onofrio N., Martino E., Balestrieri A., Mele L., Cautela D., Castaldo D., Balestrieri M.L. (2022). Diet-derived ergothioneine induces necroptosis in colorectal cancer cells by activating the SIRT3/MLKL pathway. FEBS Lett..

[72] Feng C., Yan Q., Li X., Zhao H., Huang H., Zhang X. (2025). Discovery of a Gut Bacterial Pathway for Ergothioneine Catabolism. J. Am. Chem. Soc..

[73] Marone P.A., Trampota J., Weisman S. (2016). A Safety Evaluation of a Nature-Identical l-Ergothioneine in Sprague Dawley Rats. Int. J. Toxicol..

