# Supplementary material for: Is Ergothioneine an Important Source of Plasma Trimethylamine N-Oxide in Humans?
Source: Antioxidants (Basel). 2026 Jun 29;15(7):819. doi: 10.3390/antiox15070819 (PMC13405746; doi:10.3390/antiox15070819)
Supplement: Supplementary file 1 [file antioxidants-15-00819-s001.zip › antioxidants-4319258-supplementary.pdf]

Supplementary

Table S1: Baseline characteristics of the control population (n = 12) for comparison of TMAO levels.

**Demographics**	
Age (years)	74.4 (62.8–83.2)
Female, n (%)	6 (50%)
BMI (kg/m²)	25.7 (24.1–26.7)
**Ethnicity**	
Chinese	4 (33.3%)
European	6 (50%)
Malay	0 (0%)
Maori	0 (0%)
Other	2 (16.7%)
**Laboratory values**	
Plasma creatinine (µmol/L)	82.5 (74.8–92)
**Smoking status**	
Never	8 (66.7%)
Former	0 (0%)
Current	4 (33.3%)

Values are median (interquartile range) for continuous and n (%) for categorical variables.
